# Pathways to understanding the genomic aetiology of osteoarthritis

**DOI:** 10.1093/hmg/ddx302

**Published:** 2017-07-27

**Authors:** Elena Cibrián Uhalte, Jeremy Mark Wilkinson, Lorraine Southam, Eleftheria Zeggini

**Affiliations:** 1 Human Genetics and Cellular Genetics, Wellcome Trust Sanger Institute, Hinxton CB10 1SA, UK; 2 Department of Oncology and Metabolism, University of Sheffield, Sheffield, S10 2RX, UK; 3 Wellcome Trust Centre for Human Genetics, University of Oxford, Oxford OX3 7BN, UK; 4 Human Genetics, Wellcome Trust Sanger Institute, Hinxton CB10 1SA, UK

## Abstract

Osteoarthritis is a common, complex disease with no curative therapy. In this review, we summarize current knowledge on disease aetiopathogenesis and outline genetics and genomics approaches that are helping catalyse a much-needed improved understanding of the biological underpinning of disease development and progression.

## Introduction

Osteoarthritis (OA) is the most prevalent musculoskeletal disease and a leading cause of disability worldwide (1–3). The impact of OA across Europe has been described as immense (4,5). OA affects 40% of individuals over the age of 70 (1), is a major cause of pain (6) and is associated with an increased risk of comorbidity and death (7). Ten million people suffer from OA in the UK alone, with a total indirect cost to the economy of £14.8 billion per annum (7). The most common OA site is the knee, affecting 1 in 5 people over the age of 45 (8,9). The health economic burden of OA is rising, commensurate with longevity and obesity rates (8). There is currently no treatment; disease management targets the main symptoms of pain and loss of function and culminates in joint replacement surgery [1.76 million per year in the EU (10)] with variable patient-reported outcomes (11–13). Thus, there is a large unmet need for therapeutic interventions to alter the natural history of the disease (14). This review will outline established and emerging pathways to improving our understanding of disease aetiopathology through genetics and genomics studies.

## OA Is a Disease of The Synovial Joint

The synovial joint is a complex structure, comprising articular cartilage, subchondral bone, synovial lining membrane, fibrous joint capsule and supporting ligaments. The articular cartilage, calcified cartilage and subchondral bone form the osteochondral unit, a biocomposite that is uniquely adapted to transferring loads during weight bearing and joint motion. The osteochondral unit provides tensile strength, compressive resilience and a low-friction articulating surface through the collagen network, proteoglycan aggregates and layer of lubricants, respectively. Chondrocytes are the only cell type in articular cartilage, which is avascular and aneural. Under normal physiological conditions the synovial membrane consists of a thin layer of cells with phenotypic features of macrophages and fibroblasts (15), and serves to produce synovial fluid that is responsible for maintaining nutrition and lubrication of the articular cartilage. The subchondral bone adapts its structural and functional architecture in response to its local mechanical environment through remodelling, regulated by osteocytes via interactions with osteoclasts and osteoblasts (16).

**Figure 1.**
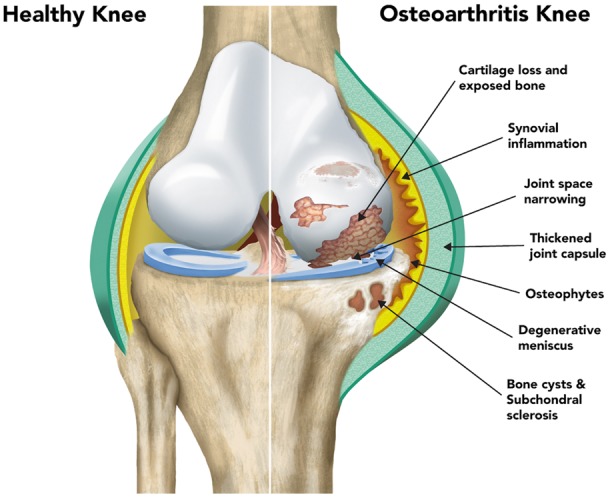
Illustration showing the key pathological features of osteoarthritis. The left side of the image shows the normal knee and the right side shows the diseased joint.

OA is a disease characterized by a gradual process of tissue destruction and remodelling that affects all of the structures of the synovial joint (17,18), with degeneration of articular cartilage, remodelling of the underlying bone, and synovitis (18) as its hallmarks (Fig. 1). The initiating signals that trigger the development of OA remain poorly understood, however established clinical risk factors include increasing age (19–21), female sex (19,21–23), obesity (20,23,24), occupational exposure to high levels of joint loading activity (23,24), previous joint injury and deformity (25,26), smoking status and family history of OA (27–30). Histological changes in OA include synovial hypertrophy and hyperplasia, with macrophage and lymphocyte recruitment, angiogenesis, and fibroblast proliferation. Those within the osteochondral unit includes loss of chondrocytes in the superficial zone with proliferation in deeper zones; loss of extracellular matrix; vascularization and neuronal ingrowth across the tidemark between calcified and non-calcified cartilage; and remodelling of subchondral bone, resulting in sclerosis, cysts and osteophyte formation.

## OA Is a Complex Genetic Disease

**Figure 2.**
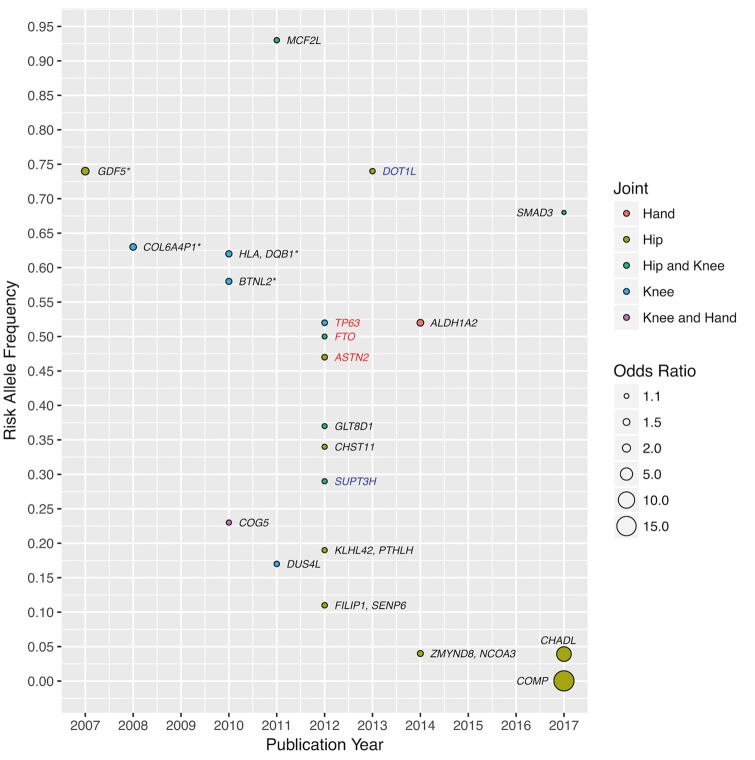
Established OA loci. Each locus is identified by the nearest gene(s) and coloured black to represent association in both sexes, blue for males only and red for females only. The discovery study population is of European descent unless indicated by an asterisk in which case the population is Asian (*BTNL2* was identified in a combined Asian and European analysis).

Both environmental and genetic factors play a role in the aetiology of OA, with genetic factors accounting for half of the variation in OA susceptibility (30). To date, and all within the last 10 years, 21 robustly established OA genetic loci have been reported (Fig. 2). With the exception of *GDF5*, which was originally identified by a candidate gene-based approach, the remaining loci were established by genome-wide association studies (GWAS). Primarily the studies were carried out in populations of European descent, with two studies performed in Asian populations and none in African. The majority of associations are joint-specific with differences in effect between end-stage and radiographic OA, as well as between males and females. Most of the variants are common (minor allele frequency >4%) with small to moderate effect sizes [largest odds ratio (OR) 1.79 for *GDF5*]. All of these characteristics typify the polygenic and complex genetic architecture underpinning OA.

A recent study of end-stage hip OA in Icelanders identified two rare variants located in the chondroadherin-like protein (*CHADL*) and cartilage oligomeric matrix protein (*COMP*) genes, with substantially larger effect sizes than previously seen (OR 7.7 and 16.7, respectively) (31). The *COMP* variant c.1141 G > C (allele frequency 0.026%) is a missense variant unique to the Icelandic population. COMP is a functional constituent and abundantly expressed in the extracellular matrix of cartilage (32), and has served as a serum biomarker for cartilage degradation (33,34). The *CHADL* variant rs532464664 (homozygote frequency 0.15%, recessive model association) is an insertion resulting in a frameshift and has been observed in other, mostly European populations. *CHADL* is expressed in cartilaginous tissue and is involved in fibrillogenesis and regulation of chondrocyte differentiation (35). Possession of these rare variants in *COMP* and *CHADL* significantly decreased the age at which total hip replacement was performed by 13.5 and 4.9 years, respectively.

Among the 21 established OA loci, several have been found to have potentially pleiotropic effects; *GDF5* is also associated with height; *FTO* with body mass index (BMI) (with *FTO* exerting its effect on OA through BMI); and a recently established OA locus in *SMAD3* with bone mineral density (BMD) (36). Furthermore, variants in astrotactin (*ASTN2*) are associated with total hip replacement in females (37) and also with migraine (38). *ASTN2* is highly expressed in the developing brain and is involved with migrating neurones. Shared pathophysiological features between migraine and OA remain unclear, although it is noteworthy that the major symptom of OA is pain. Genome-wide linkage disequilibrium regression analyses can identify genetic correlations between OA and a wide range of complex physiological, molecular and behavioural traits, followed by formal Mendelian randomization approaches to determine the direction of effect.

Several of the established OA loci also have some translational potential. Variants in the carbohydrate (chondroitin 4) sulphotransferase 11 (*CHST11*) gene are associated with hip OA (37). The protein catalyses the transfer of sulphate groups in chondroitin sulphate, the principal proteoglycan in cartilage. Chondroitin sulphate can be taken as a nutritional supplement for OA and although numerous trials have been performed the clinical benefits and pain relief evidence is inconsistent (39). Variants in the parathyroid hormone-related protein (*PTHLH*) gene are associated with hip OA (37). *PTHLH* is involved in the regulation of endochondral bone development and its analogs are prescribed for osteoporosis because of their anabolic actions on bone formation (40). Subchondral bone remodelling is also a consistent feature in OA (17,18), and may provide a novel target for intervention in OA progression.

OA cannot be considered to be a single disease. The requirement for better phenotype definition and homogeneity in much larger sample sizes is a prerequisite for untangling the genetic complexity underlying pathogenesis and progression. Electronic health records provide an excellent opportunity as they provide the ability to study large sample sizes with a wealth of clinical information. Furthermore, longitudinal information can improve phenotype definition and depth, and coupled with large scale, can improve power.

## Insights from Rare Musculoskeletal Diseases

Studying severe phenotypes of rare diseases can shed insights into the mechanisms underpinning more common disorders and identify potential therapeutic targets, e.g. the study of rare bone disorders contributed to the development of bisphosphonates (41,42). Similarly, the study of rare cartilage syndromes and developmental skeletal dysplasias can glean insights into important genes and potential targets for OA (43,44).

## Pathway to Mechanism of Disease Progression

**Figure 3.**
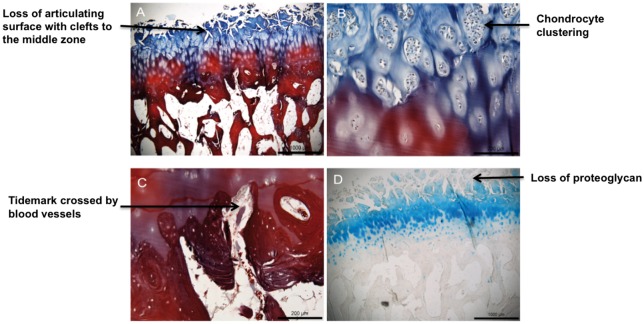
Intact and degraded cartilage-bone interface (osteochondral unit). (**A**) Cartilage/bone interface showing fibrillation and loss of articular cartilage (blue); (**B**) magnified image showing clustering of chondrocytes in the deeper cartilage layers; (**C**) magnified image of the cartilage/bone interface showing blood vessels invading the cartilage layer; (**D**) loss of proteoglycan indicated by loss of Alcian blue staining.

Unlike in most other common complex diseases, the relevant tissue for OA is readily accessible at joint replacement surgery. OA leads to changes in the joint tissue that can be captured macroscopically (Fig. 3). This provides an opportunity to study the key osteochondral unit components in order to identify signatures of disease progression. There has been great progress in understanding changes in the composition, functional properties and structure of the osteochondral unit during the evolution of OA (16,18), but it is important to also understand at the molecular level how interactions between cartilage and bone cells affect disease development.

Recent advances have signalled the development of high-resolution, high-throughput genome-wide technologies for assessing genome function, including spatial transcription (45–47), chromatin accessibility (48) and 3-dimensional conformation (49). The epigenome, transcriptome and proteome are unique for each tissue and cell type. Although systematic cataloguing of molecular maps has been carried out for many tissues (50–52), the landscape of cell types relevant to OA largely remains unknown. The accessibility of relevant tissue at joint replacement surgery enables the deployment of multi-omics to dissect the molecular disease processes in the right cells. Functional genomics is a nascent, but emerging, field in OA (53). Previous work has compared intact and degraded cartilage in modest numbers of patients (median *n =* 20) (54), investigating genome-wide methylation (55–58), gene (57–60) and/or protein expression (58,61,62), primarily through microarray technologies. Despite overall limited replicability (due to design, technology and analysis differences), pathways such as WNT signalling, angiogenesis, immune response and matrix degradation have been implicated by more than one study (59–61,63–72). The molecular architecture of genome regulation underlying disease mechanisms in OA tissue, for example as elegantly demonstrated through the study of chromatin conformation in T and B-cells in rheumatoid arthritis (73,74), remains unclear.

## Integration of Genetics and Genomic Information

Despite progress in identifying genomic regions that harbour OA-associated variants, we know very little about the specific genes involved or the way in which they mediate changes in the joint. Most of the established OA loci discovered to date (75) reside in non-coding sequence, making their biological interpretation challenging. Indeed, even though >80% of all published complex disease loci are found outside of protein-coding exons (76–78), we still have a very limited understanding of the way in which they act on disease pathogenesis (50,79–81). Identification of the causal variants and the genes they affect requires experimental analysis of genome regulation in the right cell type. Evidence is emerging from targeted studies using cell lines and patient samples that OA risk variants are likely to exert their effects on gene expression levels (82–87). Integration of genetic and genomic information is required to define molecular mechanisms through which the non-coding variants underlying association signals exert their effects on OA susceptibility (88).

## Mechanistic Studies

The molecular mechanisms linking sequence variation to cellular functions remain poorly described. To date, the characterization of OA-associated variants has mainly focused on the effect of the variant on the candidate target gene expression. Only a small number of studies have investigated the cellular and molecular mechanisms played by the identified OA susceptibility genes during OA development.

One of the best functionally characterized OA susceptibility signals is rs143383, which negatively affects the activity of the *GDF5* gene promoter, reducing the levels of *GDF5* expression (82,83,89). GDF5, a member of the BMP family and TGF-beta superfamily, plays a key role in chondrogenesis during joint development (90) and also in postnatal joint tissue homeostasis. Mice deficient in GDF5 show severe joint damage, decreased subchondral bone density and abnormal arrangement of collagen fibres in the bone (91). Also, recent studies in human primary chondrocytes have shown that GDF5 stimulation reduces the expression of the matrix degrading enzymes MMP13 and ADAMTS4 (92), both implicated in cartilage extracellular matrix degradation in OA (93,94).

Other OA candidate genes studied in cellular or animal models are *RUNX2* (rs12206662/rs10948155, associated with hip cartilage thickness and hip OA) (95) and *DOT1L* (rs1298744, rs11880992, associated with hip cartilage thickness and hip OA) (95–97). For example, knocking down the expression of Dot1l in the chondrogenic cell line ATDC5 results in reduced chondrogenesis differentiation accompanied of up-regulation of matrix metalloproteinase 9, reduced collagen content and less sulphated proteoglycans, suggesting a protective role of DOT1L in OA development (96). Knocking out Runx2 in articular chondrocytes in a mouse model of OA could rescue part of the cartilage degradation and subchondral sclerosis as well as reduce the expression of MMP13 (98).

**Figure 4.**
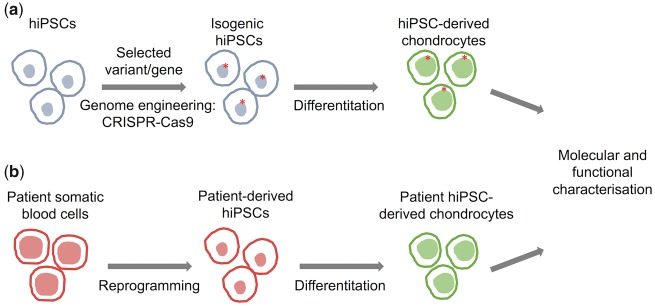
Functional characterization of loci associated with osteoarthritis. Schema showing an approach to functionally characterize OA-associated variants and candidate target genes using isogenic hiPSC derived chondrocytes (**A**) and patient hiPSC-derived chondrocytes carrying the selected variant (**B**).

Despite these recent advances, the mechanism of action of most OA susceptibility loci remains unknown. An important current effort to advance the systematic genetic, molecular and cellular functional study of genetic variation in OA is being carried out by the Origins of Bone and Cartilage Disease (OBCD) consortium, which carries out high-throughput musculoskeletal phenotyping of knockout mice (99). Cellular genetics and phenotyping models can further enhance our understanding of aetiopathogenesis. The combined use of human induced pluripotent stem cells (hiPSC) and genome editing technologies, such as the CRISPR-Cas9 (100) system, allows the targeted modification of the human genome and the generation of isogenic hiPSC lines that differ only at the specific gene or variant of interest (100–103). In the case of OA, the resulting hiPSC lines could then be differentiated towards chondrocytes (104,105), osteoblasts (106) or any other relevant cell type, creating a genetically controlled experimental model of OA. Such a model would allow the systematic study of the individual contribution of OA disease-associated variants to the disease molecular and cellular phenotype (Fig. 4). Furthermore, the possibility to derive hiPSCs from somatic cells from patients and differentiate them into disease-relevant cell types, for example chondrocytes, offers a unique opportunity to recapitulate human development and pathogenic processes.

## Pathway to New Therapies for OA

Osteoarthritis is typically diagnosed on the basis of relevant clinical symptoms and signs, and corroborated with consistent radiographic features. No robust laboratory biomarkers exist for OA. Pharmacological therapies such as paracetamol and non-steroidal anti-inflammatory drugs can be effective in relieving pain but are incapable of reversing cartilage damage (107). In the absence of a curative therapy, management strategies currently focus on interventions early in the OA joint degeneration process and targeting disease progression (18), including emerging regenerative therapies that hold the potential to promote cartilage repair and ultimately restore the original tissue structure and function. A better understanding of the molecular processes underpinning OA in the joint will be crucial to inform and accelerate the success of this new generation of treatments.

## Future Perspective

There are currently no approved disease-modifying treatements available for OA and treatment focusses on surgical replacement of the diseased joint. However, the field of OA genetics and genomics is currently witnessing an exciting alignment of opportunities to deploy a multi-pronged attack to solve the current therapeutic impasse. These approaches include massive-scale genetics with linkage to deep clinical phenotypes and patient-reported pain indices through electronic health records, access to primary disease tissue following joint replacement surgery for deep characterization of the local molecular landscape and biomarker discovery, high-throughput genome editing techniques in primary and hiPSC-derived chondrocytes to pin down causal genes, coupling joint imaging to genomics, leveraging lessons from rare musculoskeletal disorders, and emerging regenerative medicine approaches. Taken together, these technologies have the potential to alter the natural history of OA and improve the lives of these patients in the same way that the introduction of biologic treatments has changed the lives of patients with inflammatory arthritis.
